# Delayed-Onset and Prolonged Laryngeal Edema Following Pyrethroid Insecticide Poisoning: A Case Report

**DOI:** 10.7759/cureus.107257

**Published:** 2026-04-17

**Authors:** Kazutake Hasunuma, Chiaki Toida, Yasuaki Maeda, Mayo Akita, Hiroshi Imamura

**Affiliations:** 1 Advanced Emergency and Critical Care Medicine, Shinshu University Hospital, Matsumoto, JPN

**Keywords:** airway management, laryngeal edema, permethrin, pyrethroid poisoning, tracheostomy

## Abstract

Pyrethroid insecticides are generally considered to have low mammalian toxicity; however, severe toxicity may occur following high-dose or intentional ingestion. Pyrethroids are classified into two types, Type Ⅰ and Type Ⅱ, with permethrin belonging to Type Ⅰ, which is typically associated with tremor. Laryngeal edema associated with pyrethroid poisoning has been rarely reported and is usually acute and self-limited. We report a case of delayed-onset and prolonged laryngeal edema following permethrin ingestion that required extended airway management and tracheostomy.

A 73-year-old woman attempted suicide by ingesting a permethrin-based insecticide. She was intubated on admission for airway protection, showing no evidence of laryngeal edema at that time. Seizures occurred on hospital days two and three, likely attributed to pyrethroid neurotoxicity. Although her neurological status improved, extubation on day eight resulted in inspiratory stridor. Laryngoscopy revealed marked laryngeal edema, necessitating reintubation. Despite corticosteroid therapy, the edema persisted for more than two weeks, and a tracheostomy was performed on day 16. The patient was decannulated on day 30 and discharged without sequelae.

This case demonstrated that pyrethroid poisoning can induce delayed-onset and prolonged laryngeal edema, even in the absence of initial airway findings. Clinicians should remain vigilant as airway compromise may evolve beyond the acute phase; consequently, extended monitoring of the upper airway is warranted in cases of severe pyrethroid toxicity.

## Introduction

Pyrethroid insecticides, synthetic derivatives of naturally occurring pyrethrins, are widely used in household and agricultural settings. Their relatively low mammalian toxicity is attributed to rapid metabolic detoxification and lower sensitivity of mammalian sodium channels compared with insects [1]. Consequently, pyrethroids are often perceived as relatively safe.

Nevertheless, clinically significant toxicological symptoms can occur after intentional or high-dose exposure [1-3]. Pyrethroids are broadly classified into Type Ⅰ and Type Ⅱ compounds, with permethrin belonging to Type Ⅰ, which is typically associated with tremor. Neurological manifestations such as seizures are well explained, whereas severe upper airway complications are scarcely reported [4,5]. Laryngeal edema associated with pyrethroid exposure has typically been described as an acute phenomenon resolving within days.

We report a case of delayed-onset and prolonged laryngeal edema following permethrin ingestion, ultimately requiring tracheostomy.

## Case presentation

A 73-year-old woman with no recorded history of allergies or frailty was found at home with decreased consciousness and vomiting. Empty containers at the scene suggested ingestion of approximately 100 mL of a household permethrin-based insecticide, which was considered likely intentional based on the clinical circumstances. She was promptly transported to the emergency department by emergency medical services.

On admission, vital signs were stable (blood pressure 150/89 mmHg, heart rate 85 beats/min, respiratory rate 25 breaths/min, SpO₂ 95% on room air). She exhibited snoring respirations but no stridor or cyanosis. Neurologic examination revealed a Glasgow Coma Scale score of E1V1M4. Initial laboratory findings are summarized in Table 1. Endotracheal intubation was performed to protect the airway, and no laryngeal edema was observed at the time of intubation.

**Table 1:** Laboratory findings of the patient experiencing permethrin poisoning at admission. Abbreviations: eGFR, estimated glomerular filtration rate; AST, aspartate aminotransferase; ALT, alanine aminotransferase; LDH, lactate dehydrogenase; ALP, alkaline phosphatase; CRP, C-reactive protein; γ-GTP: gamma-glutamyl transpeptidase; PT-INR, prothrombin time–international normalized ratio; APTT, activated partial thromboplastin time. * This elevated eGFR likely reflects low serum creatinine rather than true hyperfiltration.

Parameter	Value	Reference Range
White Blood Cell	16.29	4.0 – 10.0 ×10³/μL
Hemoglobin	13.2	13.0 – 17.0 g/dL
Hematocrit	41.8	39 – 52 %
Platelet Count	241	150 – 400 ×10³/μL
Sodium	145	135 – 145 mmol/L
Potassium	2.8	3.5 – 5.0 mmol/L
Chloride	107	98 – 108 mmol/L
Calcium	9.0	8.5 – 10.5 mg/dL
Magnesium	2.2	1.6 – 2.6 mg/dL
Phosphate	3.2	2.5 – 4.5 mg/dL
Blood Urea Nitrogen	11.4	8 – 20 mg/dL
Creatinine	0.29	0.6 – 1.2 mg/dL
eGFR	162^*^	> 60 mL/min/1.73m²
Glucose	148	70 – 110 mg/dL (fasting)
AST	21	10 – 40 U/L
ALT	7	5 – 45 U/L
LDH	294	120 – 245 U/L
ALP	128	100 – 340 U/L
γ-GTP	18	10 – 50 U/L
Total Bilirubin	1.20	0.2 – 1.2 mg/dL
Direct Bilirubin	0.36	0.0 – 0.4 mg/dL
CRP	0.44	< 0.3 mg/dL
Creatine Kinase	130	55 – 170 U/L
Troponin T	0.010	< 0.014 ng/mL
PT-INR	1.18	0.9 – 1.1
APTT	46	25 – 35 sec

**Figure 1:**
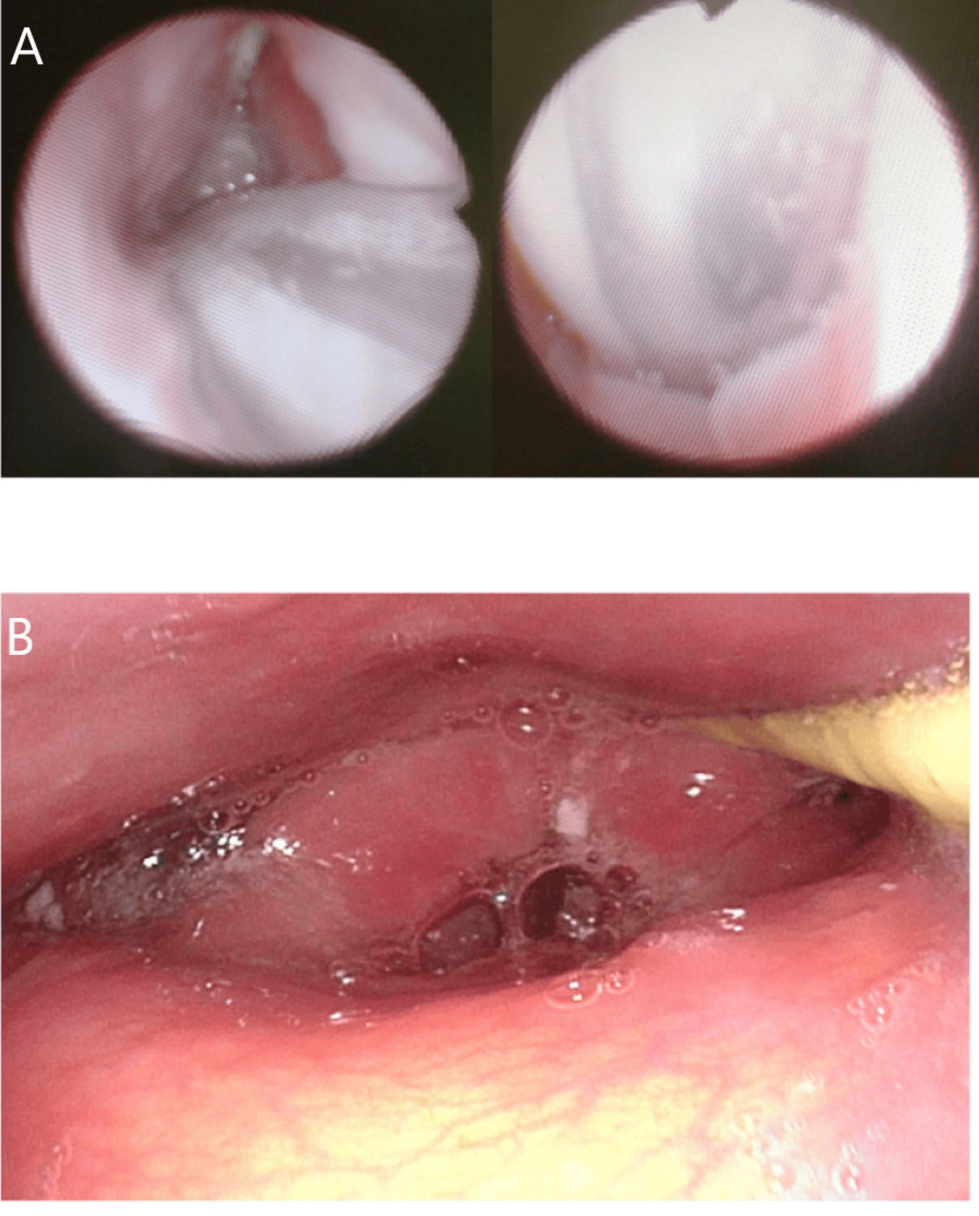
Laryngoscopy imaging of the patient experiencing permethrin toxicity. (A) Laryngoscopy image on day 13 of illness (B) Laryngoscopy image on day 26 of illness Persistent laryngeal edema is observed on both day 13 (A) and day 26 (B). Salivary pooling is visible after tracheostomy.

Supportive therapies, including activated charcoal administration, were initiated. On hospital days two and three, the patient experienced generalized seizures attributed to pyrethroid neurotoxicity. By day six, neurological status improved, but a cuff leak test was negative, suggesting airway edema. Corticosteroids were administered. Despite a negative cuff leak test suggesting possible airway edema, extubation was attempted based on overall clinical improvement, including recovery of consciousness and stable respiratory status. After clinical stabilization, extubation on day eight resulted in inspiratory stridor within 30 minutes. Laryngoscopy demonstrated marked laryngeal edema with impaired vocal cord motion. Reintubation was required. Repeat laryngoscopy on day 13 revealed persistent edema, and tracheostomy was performed on day 16 due to prolonged airway compromise (Figure 1A).

The patient was released from mechanical ventilation on day 20. On day 26, the edema showed a tendency to improve but persisted, with decreased reflexes and retention of saliva observed (Figure 1B). Her condition gradually improved, she was decannulated on day 30, and discharged on day 36 in stable condition.

## Discussion

This case describes a rare but clinically significant manifestation of pyrethroid insecticide poisoning: delayed-onset and prolonged laryngeal edema requiring tracheostomy. Although pyrethroids are recognized to have low mammalian toxicity, this case demonstrates that serious and atypical airway complications can occur following intentional ingestion [1,2].

Pyrethroid poisoning most commonly manifests with gastrointestinal and neurological symptoms such as nausea, vomiting, agitation, tremor, and seizures [1,2]. These symptoms are triggered by prolonged activation of voltage-gated sodium channels, resulting in neuronal hyperexcitability [3]. Respiratory complications are less frequently reported and typically include pulmonary edema or respiratory failure [1,2]. Upper airway involvement, particularly laryngeal edema, has been reported only sporadically and is usually acute in onset, developing within hours of exposure and resolving within several days after corticosteroid therapy [4,5].

In contrast, this patient exhibited a distinctly different clinical course. No laryngeal edema was observed during initial intubation, and the airway appeared stable during the early phase of hospitalization. Laryngeal edema became clinically evident more than one week after ingestion and persisted for over two weeks, ultimately necessitating tracheostomy. This delayed onset and prolonged duration distinguished this case from previously reported pyrethroid-associated airway complications (Table 2) [4,5].

**Table 2:** Comparison of the present case study on permethrin poisoning with the past cases. Abbreviations: IV, intravenous.

Case No.	Age/gender	Exposure Route	Active Pyrethroids Ingredient	Respiratory symptoms	Time to onset	Airway management	Other treatment	Time to resolution	Outcome
Current Case	73/ female	Oral ingestion and inhalation	Permethrin	Laryngeal edema	5 d	Intubation, reintubation, tracheostomy	IV methylprednisolone	>14 days	Recovered Discharged on Day 36
Case 1[4]	78 / male	Inhalation	Tetramethrin, Pyrethrin and Phenothrin	Laryngeal edema, throat discomfort, dyspnea	24 h	Intubation, tracheostomy	IV methylprednisolone	<8 days	Recovered Discharged on Day 12
Case 2 [5]	52 / female	Inhalation	Permethrin	Dyspnea, stridor	<1 h	Oxygen	IV hydrocortisone, midazolam	<1 day	Recovered Discharged next day
Case 3 [5]	63 / male	Inhalation	Permethrin and Cyphenothrin	Dyspnea, stridor, cough, eye/throat irritation	<1 h	Oxygen	supportive care	<1 hour	Recovered without admission
Case 4 [5]	20 / male	Inhalation	Permethrin	Dyspnea	5 h	Oxygen	IV methylprednisolone 1 g	<1 day	Recovered Discharged next day

Several mechanisms may explain this atypical presentation. Alterations in metabolic pathways, including delayed clearance or accumulation of pyrethroid metabolites, may have contributed to the prolonged clinical course observed in this case [1,2]. In addition, sustained activation of voltage-gated sodium channels may have induced prolonged neuronal hyperexcitability and neurogenic inflammation, potentially contributing to delayed airway mucosal edema [1,3]. Furthermore, solvent components in the ingested formulation may have exacerbated mucosal injury and inflammatory responses [2,6].

Pyrethroids are primarily metabolized in the liver via cytochrome P450 (CYP) enzymes [2], and age-related reductions in hepatic metabolic capacity may delay the clearance of both parent compounds and their metabolites. In this elderly patient, such pharmacokinetic alterations may have prolonged systemic exposure and contributed to the delayed onset and persistence of airway inflammation.

These mechanisms may have acted synergistically, resulting in the delayed onset and prolonged duration of laryngeal edema observed in this patient.

The estimated oral exposure approached the lower range of reported oral LD₅₀ values in animal models [7,8]. Although ingestion was the primary exposure route, the circumstances of exposure - application in an enclosed space, subsequent vomiting, and delayed discovery - highlight the possibility of concomitant inhalational exposure. Synergistic effect on exposure routes may therefore have amplified airway toxicity and contributed to the severity and prolonged nature of the laryngeal edema [1,2,5].

Post-intubation laryngeal edema was considered but deemed unlikely. No laryngeal edema was observed at the time of intubation, and airway findings remained stable during the early phase. The edema became clinically apparent only after several days, suggesting a delayed onset. Known risk factors include traumatic or prolonged intubation, inappropriate tube size, excessive cuff pressure, fluid overload, malnutrition, and allergic predisposition [9]. Although a negative cuff leak test on day six suggested possible airway narrowing, this finding alone is not specific for clinically significant laryngeal edema and does not explain the delayed progression and prolonged course. In this case, endotracheal tube size was considered appropriate, cuff pressure was monitored, and no systemic edema or fluid overload was observed. Furthermore, edema persisted even after tracheostomy, which is atypical for post-intubation laryngeal edema. These findings support a toxicologic rather than mechanical etiology.

From a medical toxicology perspective, this case has several important implications. Airway complications following pyrethroid poisoning may evolve independently, irrespective of early clinical findings, and may extend beyond the acute toxic phase. Readiness for extubation should be assessed cautiously, and adjunctive evaluations such as cuff leak testing and early laryngoscopy may be specifically valuable [9]. Clinicians should maintain a low threshold for prolonged airway surveillance and be prepared for reintubation or tracheostomy in cases of severe exposure.

To the best of our knowledge, this is a rare case of pyrethroid-associated laryngeal edema persisting for more than two weeks and requiring tracheostomy. This case advances the spectrum of recognized pyrethroid toxicity, underscoring the need for vigilance for delayed and prolonged airway complications, particularly in intentional ingestions or scenarios involving combined exposure routes [1,2,4-6].

## Conclusions

Permethrin poisoning can cause delayed-onset, persistent laryngeal edema warranting prolonged airway support and tracheostomy. This case underscores the importance of vigilant monitoring for atypical airway complications in pyrethroid exposures.
